# Data sharing for zoonoses surveillance in Senegal: challenges and opportunities

**DOI:** 10.3389/fvets.2026.1739586

**Published:** 2026-02-02

**Authors:** Khady Diouf, Marion Bordier, Jean Hugues Caffin, Assane Gueye Fall, Mame Thierno Bakhoum, Thomas-Julian Omoijade Irabor, Nicolas Antoine-Moussiaux, Rianatou Bada-Alambedji

**Affiliations:** 1 National Laboratory for Livestock and Veterinary Research, Senegalese Institute of Agricultural Research, Dakar, Senegal; 2 Department of Immunology and Infectious Pathology (Microbiology), Interstate School of Veterinary Science and Medicine, Dakar, Senegal; 3 Doctoral School of Life Sciences, Health and Environment, Université Cheikh Anta Diop de Dakar, Dakar, Senegal; 4 ASTRE, University of Montpellier, CIRAD, INRAE, Montpellier, France; 5 CIRAD, UMR ASTRE, Dakar, Senegal; 6 Institute of Health and Development, Université Cheikh Anta Diop de Dakar, Dakar, Senegal; 7 Fundamental and Applied Research for Animals and Health, University of Liege, Liege, Belgium

**Keywords:** data, governance, one health, Senegal, surveillance, zoonoses

## Abstract

**Introduction:**

Zoonoses are a growing threat to global health, requiring effective integrated surveillance. Such surveillance relies on the structured and secure sharing of data between human, animal and environmental health stakeholders. In Senegal, despite advances in the One Health approach, surveillance data sharing remains limited. This study aims to analyze challenges related to surveillance data sharing in Senegal and identify ways to improve the situation.

**Methods:**

This study adopted an inductive qualitative approach to explore the challenges and opportunities of data sharing through 61 semi-structured interviews with three categories of key stakeholders: (i) system managers, (ii) initiative holders, and (iii) community actors. A thematic analysis was applied to the participants’ discourse to identify the main issues and opportunities for improvement. Data were triangulated by cross-referencing interview contents with institutional documents and previous studies to ensure the robustness of the results.

**Results:**

Three significant challenges are holding back data sharing for zoonosis surveillance: (i) limited technical and organizational capacities, which compromise interoperability, accessibility, and data utilization; (ii) contrasting attitudes among stakeholders, between motivation and mistrust or reluctance, linked to transparency and recognition concerns; and (iii) fragmented governance, characterized by the absence of a clear regulatory framework, a lack of coordination, and insufficient sustainable funding. However, opportunities exist, including stakeholder motivation when collaboration is recognized and rewarding, and existing institutional frameworks that could be strengthened.

**Conclusion:**

To advance data sharing and strengthen zoonotic surveillance in Senegal, the study highlights the necessity to harmonize existing surveillance systems, reinforce capacities, and establish collaborative governance. These efforts should address stakeholders’ concerns while supporting their motivation to contribute to an integrated surveillance system.

## 1 Introduction

Globalization and intensification of international exchanges have considerably increased health risks, particularly those related to zoonotic diseases. These diseases, transmissible between animals and humans, represent a growing threat to global public health. According to the World Health Organization (WHO), nearly 60% of human infectious diseases are of zoonotic origin. The emergence of these pathologies is closely linked to factors such as environmental change, rapid urbanization and international trade, which favor the dispersal of pathogens across borders (1).

In Senegal, as well as in West Africa, zoonoses pose serious health and economic challenges. Studies have highlighted the impact of zoonotic diseases such as avian influenza, Rift Valley Fever, rabies, and hemorrhagic fevers (Marburg and Ebola), which affect not only public health but also people’s livelihoods (2, 3).

Epidemiological surveillance is based on the systematic and continuous collection of data to monitor the health status and risk factors of a defined population, to compile and analyze them, then disseminating timely information that contributes to the planning, implementation and evaluation of risk management measures (1, 4). Surveillance of zoonotic diseases requires establishing integrated surveillance systems that bring together surveillance programs operating in human, animal and environmental sectors, to improve the information collected and its use for better health management (5). Zoonotic disease management, therefore, requires the implementation of systemic approaches based on collaboration between the different sectors and professions involved in their prevention and control, and communities, following the One Health approach (6, 7).

In Senegal, this approach is based on close collaboration between the Ministries of Health, Livestock, and Environment, coordinated by the One Health National High Council for Global Health Security (NHCGHS-OH), also usually called “One Health Platform.” Created in 2017, this body is under Prime Minister’s Office authority and is supported by technical and financial partners (2).

**Figure 1:**
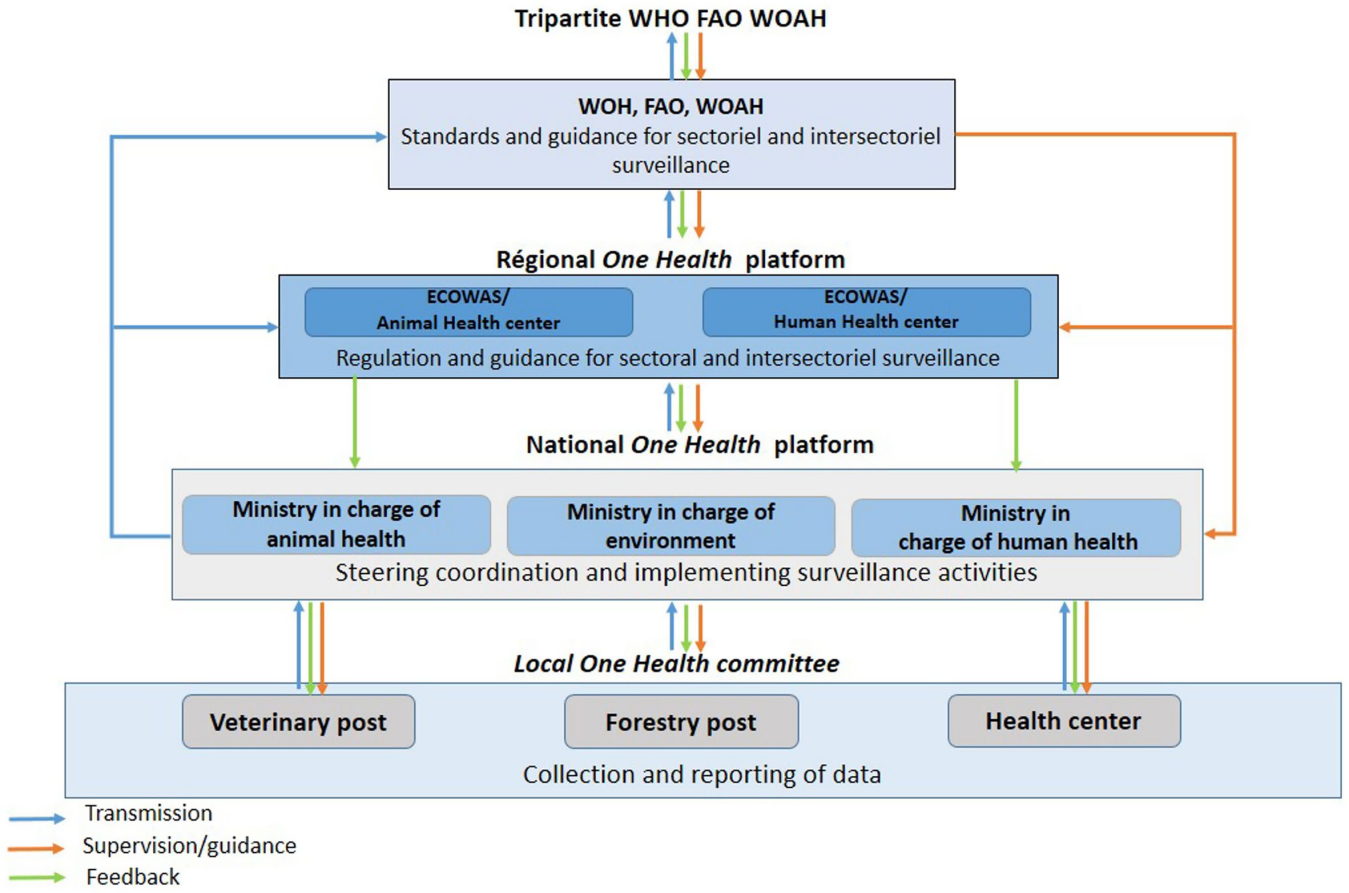
The public governance framework of surveillance for zoonotic diseases in Senegal.

The country’s governance structure adds complexity to surveillance coordination. In 2013, Senegal underwent administrative decentralization, establishing regions, departments, and communes as autonomous entities with elected councils (8). However, this decentralized system does not fully extend to disease surveillance. Surveillance activities remain largely centralized and are organized through sectoral ministries at the national level. This creates a hybrid governance model where vertical data flows follow hierarchical pathways from central to peripheral levels within each ministry, while horizontal coordination between human health, animal health, and environmental sectors requires dedicated cross-ministerial mechanisms (9, 10). At subnational levels, surveillance is implemented through decentralized sectoral services operating at regional, departmental, and local levels, which report to their respective ministries. While this pyramidal structure ensures broad geographical coverage, it can create bottlenecks in information flow. Data transmission often faces delays as information moves up hierarchical chains, and cross-sector communication within the same administrative level remains challenging (11). For zoonosis surveillance, the One Health approach underlines particularly the need for information and data sharing between different stakeholders, sectors, and between scales of operation, to enable a rapid response in case of suspected case detection and to guide appropriate risk management measures (11). In Senegal, the institutional arrangements established to govern zoonotic surveillance at international, regional, and national levels are presented in Figure 1. However, despite efforts and strong advocacy from international community to establish integrated information systems (IIS), data sharing between sectors remains limited, both vertically and horizontally. Data from universities and research institutes are not shared or are insufficiently mobilized to guide an optimal and evidence-based public decision-making. Several initiatives have already attempted to create standards to ensure interoperability between sectoral information systems (12). However, to date, no sustainable and routinized mechanism for data and information sharing is implemented.

This study examines data sharing for zoonotic surveillance in Senegal across all major categories of zoonotic diseases, including endemic, epidemic-prone, and emerging infectious diseases. Rather than focusing on specific pathogens, the analysis adopts a cross-cutting approach centered on data-sharing practices applicable to these categories within a One Health framework. It aims to provide strategic guidance to policy-makers and practitioners in improving cross-sectoral collaboration and inform the development of an integrated information system that supports a more effective One Health response to zoonotic threats.

## 2 Materials and methods

This study adopted a qualitative approach to explore data and information sharing between different stakeholders. This approach allows us to explore the problems by fully understanding the participants’ perceptions, experiences, and expectations regarding data access, use, and collaboration (13). These insights helped identify concrete challenges and opportunities for improving sustainable and integrated data sharing across sectors.

### 2.1 Data collection

A preliminary literature review and stakeholder mapping were carried out to identify key stakeholders involved in zoonosis surveillance in Senegal. This initial list was completed by members of the “Zoonosis” and “Data Management” thematic groups of the One Health Platform considering their involvement in zoonosis surveillance activities. During interviews, respondent-informed sampling was used to identify additional relevant stakeholders based on the initial stakeholders’ knowledge.

Identified stakeholders were divided into three categories: (i) system managers responsible for surveillance data collection and management (veterinary institutions, human health institutions, environmental institutions, laboratories and research institutions); (ii) initiative holders involved in programs or projects indirectly supporting surveillance data sharing (national and local authorities, international organizations, NGOs and some private actors); and (iii) community actors (local stakeholders who play a key role in data collection and transmission, such as livestock farmers, community health workers and local organizations involved in health management).

Stakeholders were included if they met at least one of the following criteria: (i) active involvement in zoonosis surveillance activities in Senegal, including data collection, management, or analysis; or (ii) generation or management of data used or potentially useful for surveillance purposes; and (iii) willingness to participate and availability during the study period (June to September 2023). These broad criteria were intentionally designed to capture the full diversity of actors involved in surveillance data flows. We did not apply rigid exclusion criteria but instead used respondent-informed sampling to progressively identify stakeholders whose roles proved most relevant to data sharing practices. Sampling continued until thematic saturation was reached. Semi-structured interview guides were elaborated to examine three main themes: (i) stakeholders’ activities in relation to zoonosis surveillance, (ii) their experience on data and information sharing, (iii) their expectations for advancing data and information sharing practices. The complete semi-structured interview guides, conducted in French and including both original versions and English translations, are provided in Supplementary material. These guides cover multiple aspects of zoonotic surveillance systems; this manuscript presents findings related to data sharing practices, challenges, and opportunities.

All participants were contacted by e-mail or telephone. Invitations were attached with an information note on the objectives of the study.

Interviews lasted 60 to 90 min. Participants, particularly community actors, were given the choice to be interviewed in Wolof (The most widely spoken local language) or French (the official administrative language). Interviews were recorded after participants consent and then fully transcribed. Institutional documents and other sources of information were collected in addition to enrich literature reviews.

### 2.2 Data analysis

Initially, all interview recordings were coded according to the participant’s category, sector, activities and action level.

Data analysis was carried out using a thematic approach, following Braun and Clarke (14) methodology. This method provided a semi-structured framework for identifying, analyzing and interpreting recurring themes in qualitative data (14).

For thematic analysis of interviews, a deductive framework, informed by the study objectives, was introduced as a guide for the coding. The framework was supplemented inductively during data analysis by themes emerging from stakeholders’ narratives, indicating the recurrence of certain experiences and considerations among the respondents.

The first step involved familiarizing ourselves with the data by carefully reading the transcripts to fully understand their content. The data were then manually coded and analyzed using Microsoft Excel. The coding process consisted of identifying relevant excerpts mentioned by participants regarding information and data sharing and ascribing them a code summarizing its topic. These excerpts were then contextualized using information about the participants’ sector, the activities they were involved in, and their level of intervention, in order to better understand the diversity of perspectives across institutional profiles.

Once the initial coding was completed, similar codes were clustered together to identify themes and sub-themes. The codes/themes/sub-themes were reviewed and gradually refined to ensure they accurately reflected the data and the specificity of excerpts while meaningfully expressing patterns across different excerpts. Each theme is precisely defined and named to capture its essence. Data saturation was assessed through iterative analysis during data collection. Saturation was considered achieved when no new themes, patterns, or insights emerged from consecutive interviews. We monitored saturation both within each stakeholder category (system managers, initiative holders, and community actors) and overall across all participants. Saturation was reached in each category, with additional interviews conducted to confirm the stability of findings.

Two researchers conducted thematic analysis. One is a Senegalese doctoral student in participatory epidemiology who is familiar with the local context. Another is a researcher specializing in integrated approaches to health who brought an external perspective and complementary expertise. The results, including themes, codes, and associated verbatims, were compared between the two researchers to ensure the validity of conclusions (15). All differences were discussed, leading to further refinement and harmonization of the understanding and labelling of results.

Finally, data triangulation was carried out by cross-referencing information from interviews with institutional documents and previous studies, which aligned with the methodology of Carter and colleagues (16). This process helped verify the consistency of stakeholder’s accounts with documented institutional practices, identify recurring issues across sources, and enrich the interpretation of key themes.

### 2.3 Ethical approval and agreement to participate

This study was evaluated and validated on June 2024 by the Health Research Ethics Committee of Ministry of Health and Social Action in Senegal (deliberation N°0000162/MSAS/CNERS/SP). Written informed consent was obtained from interviewees. The anonymity of participants and the confidentiality of data were also respected.

## 3 Results

A total of 61 actors were identified and invited to take part in the study. More specifically, 41 system managers were interviewed, divided into 13 participants at the central level, 27 at the local level and one participant at the international level. Community actors were represented by 7 local participants for 7 interviews. Initiative holders participated in 13 interviews, involving 3 central, 4 international and 6 local participants. Participants come from different sectors, including human health (22), animal health (15), environment (11), agriculture (4), and multisectoral domain (9) (Table 1).

**Table 1:** Characteristics of study participants (*n* = 61).

Participant category	Intervention level (number of participants)	Total
System managers	Central level: 13 Local level: 27 International level: 1	41
Community actors	Central level: 1 Local level: 6	7
Initiative holders	Central level: 3 Local level: 6 International level: 4	13
Total		61

“Intervention level” refers to the organizational level at which participants operate.

Numbers indicate the distribution of participants by category and intervention level.

Three main dimensions influencing data sharing in Senegal were identified: technical and organizational capacities, stakeholder positions, and inter-sectoral governance. For each category, both challenges limiting effective data exchange and opportunities for improvement are examined. These are described below with illustrative excerpts from participants to provide a comprehensive understanding of data sharing practices for zoonotic surveillance in Senegal.

### 3.1 Technical and organizational capacities

#### 3.1.1 Data sharing tools and technologies

The diversity and fragmentation of data collection and sharing tools across sectors reflect unequal technical capabilities. Methods used vary depending on the sector. For human health, platforms such as the District Health Information Software 2 (DHIS2) and Teranga are used, while animal health relies on KoboToolbox and SYLAB. However, environment, sanitation, and agriculture sectors lack formal data collection systems, which hinders data centralization.

In rural areas, this situation is compounded by contrasting realities where the tools and systems for data collection and sharing are often deemed insufficient, particularly in animal health and environment sectors. Limited internet access in rural areas further complicates data sharing. A system manager for animal health sector mentioned, for example: *“I collect information with my phone and visit a cybercafé to print my report before it is submitted to national office.”*

Private sector and research institutes have high-quality databases, but these are rarely integrated into national surveillance systems. An initiative holder from private animal health sector expressed frustration: *“I collect the data on my own; the authorities have not provided me with any equipment and have no idea about these data.”*

Similarly, initiative holders, such NGOs, support and fund zoonotic surveillance-related projects that generate valuable data. However these data are rarely integrated into the national surveillance system. In addition, these actors often lack access to government databases. Consequently, each sector uses its own tools, as no standardized data collection and centralization system has been established.

In response to these challenges, some actors have developed adaptation strategies. Meetings are organized by central and local authorities to encourage data sharing, but they are described as irregular and ineffective due to the lack of appropriate mechanisms. In environmental and agricultural sectors, data are recorded in Microsoft Excel files. At the same time, a dual reporting system is being implemented for human and animal health, combining digital platforms and physical reports, as explained by a system manager in human health sector: *“We save data on digital platforms in addition to physical reports, which ensures data availability even when we go on a strike.”*

Furthermore, most stakeholders expressed their desire to see standardization of data collection systems to facilitate sharing between sectors. A system manager in human health sector emphasized: *“We need to harmonize the collection and centralization of our data if they have to be interconnected.”* Initiative holders deplore the lack of communication between systems in different sectors. They emphasize the need for interoperability to provide an overview and pool efforts to provide evidence-based recommendations.

#### 3.1.2 Data utilization capacity

Data utilization capacity varies from one stakeholder to another. Some central-level system managers are highly skilled in data analysis, but many local stakeholders feel inadequately prepared. This inequality creates bottlenecks due to limited number of skilled persons required to interpret all the data, slowing down the decision-making process; as clearly stated by a system manager in human health sector: *“We cannot even use our own data. We do not need a data warehouse, we need operable information.”* This situation is compounded by work overload and a lack of human resources capable of exploiting the data, which increases challenges, particularly at local level, as a human health system manager noted: *“I’m overwhelmed: I do consultations, fill out forms, and at the same time fill out DHIS2 platform; I cannot do everything.”* Frequent posting of local staff exacerbate the situation, and each newly assigned agent must reacquire skills and procedures that are often insufficiently documented.

Training is organized by authorities, with NGOs support, but it remains rare and inaccessible, particularly for private and environmental sectors. As environmental system manager stated: *“There is a lack of awareness of the importance of the data we collect and their usefulness for other sectors involved in zoonotic disease surveillance; we need to be more involved in the activities, particularly in training.”*

Similarly, many system managers are showing a growing interest in strengthening their data analysis skills. Some are adopting empowerment strategies, such as seeking additional training or implementing simplified tools for data visualization. These efforts, although scattered, demonstrate a desire to improve data use. As one local system manager from environmental sector put it: *“If we are supported, we can learn how to transform our data into useful information for our work and for other sectors.”*

### 3.2 Stakeholders’ attitudes toward data sharing

Data sharing is not a technical issue alone but also a matter of trust, transparency, and mutual recognition.

#### 3.2.1 Stakeholder reluctance toward data sharing

Trust and transparency in data sharing remain major challenges for system managers. Some stakeholders fear that their data will be used for purposes other than public health, but for political or personal interests. These concerns are particularly expressed by professionals working in laboratories, research institutions, and environmental agencies. One laboratory system manager emphasized: *“I’m going to be very careful about sharing data between colleagues. Things need to be clear and depends on the data you can share.”*

The lack of clarity in the data sharing process is frequently mentioned. Stakeholders emphasize the need to establish precise guidelines before considering data integration. Without clear foundations, harmonized access to data remains difficult, compromising responsiveness to health threats. A human health system manager emphasized this: *“Before we agree to share our data in an integrated information system, we need to understand everything and work on a clear and transparent basis.”*

Community actors lament the lack of transparency regarding data use. The lack of feedback fuels community actors’ frustration and weakens their motivation to participate in surveillance activities. They advocate for regular inter-sectoral forums to strengthen collaboration.

Some system managers perceive data as a direct source of decision-making power. From their perspective, sharing information would weaken their strategic position, as they closely link control over data to influence over decisions. The logic of withholding information is based on the belief that being the one who holds the data means being better informed, and therefore more legitimate in decision-making. A human health system manager stated: *“I cannot deliberately share my data. When you have the data, you have decision-making power because you are well informed.”* The fear of losing control over the use of shared data also reinforces this reluctance. According to one epidemiologist system manager: *“I cannot collect my data and hand it over to someone without having an idea of what will be done with them. I need to be involved and know the results.”* This expressed need for involvement reflects a search for ownership and recognition, which could be better addressed through the establishment of formal feedback.

In research sector, system managers are hesitant to share their data before completing their publications. One system manager in this sector expressed his concern: *“Before sharing my data, I have to complete my publications. Nothing protects me, and if my data are used before me, what can I do?”* However, some collaborative research projects include agreements that secure publications. This represents an acceptable compromise for many researchers and allows for progressive data sharing.

Other stakeholders, particularly in the research sector, fear that sharing their data could lead to intellectual dispossession. They worry that others perhaps more skilled or visible could exploit the data without involving them or acknowledging their contribution. This concern reflects an insecurity tied to competition in scientific or institutional spaces. A research manager stated, *“Data are gold, they are power. I can have my data without being able to exploit them. If I share them with someone more talented, they can use them without collaborating with me.”*

Furthermore, some view data as an investment that cannot be given away for free, as clearly stated by a system manager from the environmental sector: *“We spend a lot of money to obtain our data, so we cannot give them for free; we sell them.”*

Community actors and local system managers are more willing to share their data than central stakeholders. However, a lack of recognition and insufficient technical and financial support discourage them. Some local community actors or system managers, however, have managed to strengthen their involvement through projects with regular feedback that values their contribution.

#### 3.2.2 Stakeholder motivation for data sharing

Many stakeholders also express motivations to share their data, particularly when this sharing is part of a collaborative and rewarding approach. System managers are more inclined to share their data when it contributes to better inter-sectoral coordination and the development of effective public policies. The commitment of system managers is often based on recognition of their contribution, the existence of ethical safeguards, and feedback mechanisms. A human system manager stated: *“We know our information can save lives, and that’s why we want to share it.”*

However, they emphasize the need to show gratitude and acknowledge their effective participation to reaffirm their commitment. Thus, recognition and appreciation of data sharing by authorities and peers encourage continued collaboration between stakeholders. A Ministry of Agriculture officer explains that this can improve interventions, which is confirmed by the words of a system manager in animal health sector who states: *“When our sharing efforts are recognized, our data are used to improve interventions, it encourages us to continue in this direction.”* Although researchers prioritize scientific publication, they hope their data also contribute to evidence-based public health knowledge.

Community actors, for their part, deplore their working conditions. They feel exposed to the risk of zoonotic diseases because they work without protective equipment. They denounce the fact that their salaries are not paid by the government but are solely supported by project funds. However, they remain motivated by the constant gratitude of their communities towards them. This fosters the feeling of being part of a solidarity chain. A community actor in human health sector states: *“We are asked to produce data without any support or motivation, even though we are not obliged to share it. ““It’s our community that gave us a mandate, and that’s what motivates us.”* This social mandate, although informal, thus appears as a strong source of motivation for community actors.

Furthermore, cultural and organizational differences also affect stakeholders’ motivation to share data. While research system managers lament the lack of proactivity and innovation on part of public authorities, the latter highlight the low level of involvement of researchers and initiative leaders in surveillance activities. One researcher denotes as a broad conclusion: *“It’s sometimes difficult to combine our different working methods.”*

Despite these tensions, several stakeholders emphasized the value of building stronger intersectoral dialogue. They argue that increased communication could foster mutual understanding and help each sector better appreciate the others’ constraints, paving the way for more effective collaboration.

### 3.3 Inter-sectoral surveillance governance

#### 3.3.1 Governance mechanism

According to system managers, the One Health Platform has contributed to improving collaboration between different sectors by formalizing existing mechanisms or creating a consultation framework conducive to interaction. However, despite these efforts these stakeholders decry the current system still operates largely in institutional silos and is characterized by a lack of harmonized data governance mechanisms, both within and between sectors. Imbalanced power relations between sectors are also reported. Explicitly, human health sector is described as being at an advantage over animal health and environmental sectors, which often lack the resources to address their own priorities.

In this context, human health system managers are calling for inter-sectoral coordination, but this request is contested by other sectors. A system manager from environment sector emphasized actors’ autonomy: *“Ministry of Health is independent, as it is for Ministry of Livestock; neither can coordinate the other’s activities. By the way, One Health Platform plays a vital role because it sits between ministries and in Prime Minister’s Office.”* Indeed, most system managers recognize that the One Health Platform, under the authority of Prime Minister’s Office, represents a strategic opportunity to structure inter-sectoral governance of data sharing. However, they emphasized the need to increase efforts to formalize the process to facilitate the smooth collaboration between different sectors. Several stakeholders lamented the lack of a regulatory framework to support data exchange between sectors and emphasized the need to clarify roles and responsibilities. A system manager from human health sector explained: *“I have no idea about the collaboration procedures for sharing my data with other sectors, even though I know it will be useful to other stakeholders.”* Similarly, an animal health system manager laments: *“Main constraints to integrated data sharing lies in the lack of a framework that clearly defines which data should be shared, by whom? With whom? And under which conditions?”*

Some stakeholders also mention that the One Health Platform must be supported by an inter-sectoral technical structure to facilitate efficient and structured use and sharing of data. A system manager in animal health sector emphasized: *“For inter-sectoral data sharing, we need to create a national technical public health institute that brings together all sectors to support the One Health Platform.”*

#### 3.3.2 Resources

Lack of human and financial resources for inter-sectoral coordination constitutes a major obstacle to effective implementation of integrated data sharing.

The stakeholders from the One Health Platform indicated that most of their activities are funded by donors. They emphasized not having a specific budget to support integrated surveillance activities or data sharing. The permanent secretariat staff of the One Health platform are seconded by various ministries. This lack of resources compromises the ability to implement continuous and sustainable actions. In the absence of structuring public investment, inter-sectoral governance appears to rely heavily on the priorities and agendas of technical and financial partners (TFPs). These actors include intergovernmental and international organizations, bilateral cooperation agencies, and international NGOs. As they provide most of resources necessary for collaboration, stakeholders concerns about long-term dependency. A system manager in animal health sector emphasized: *“We must not have national activities that depend 90% on external funding.”* A system manager in human health sector stated: *“The government must allocate budgets to departments responsible for integrated data management to strengthen surveillance systems rather than relying on financial partners.”*

Dependence on external funding is also mentioned as a source of discontinuity. According to the interviewed stakeholders, donors often fund vertical programs and target specific diseases, especially in human health sector. Collaboration is developed project by project without being incorporated into an overall strategic plan.

According to initiative holders, several data-sharing projects have stopped activities upon funding cessation because they were not integrated into a sustainable institutional strategy. They support the idea that political will is necessary to maintain collaboration between surveillance stakeholders and ensure sustainable data sharing. An initiative holder from NGO stated: *“We’ve worked a lot on integrated data sharing, and always with the same stakeholders. We need political will to capitalize on all these initiatives and build an integrated and sustainable data sharing system.”*

Although dependence on external funding creates a form of uncertainty, according to system managers, it also represents a window of opportunity. These stakeholders argue that support for donors must be better structured, managed locally, and politically driven to support government initiatives.

## 4 Discussion

This study highlights critical issues related to data sharing for zoonotic disease surveillance in Senegal. These critical issues are mainly technical and organizational skills of stakeholders, their posture and the inter-sectoral governance. However, these issues are tightly interconnected and mutually reinforcing. The need for inter-sectoral governance emerges as a key structuring element. As also discussed by Nana et al. (17), our findings suggest that governance can qualitatively and directly influence stakeholders’ means, skills and knowledge, resulting in an improvement of governance mechanisms. This dialogue or feedback loop linking an enabling governance structure and the capacities of stakeholders, gradually shape their motivation to invest and commit to collaboration (17).

### 4.1 Accessibility and use of surveillance data

The results reveal major constraints that hinder the integrated sharing of zoonotic disease surveillance data in Senegal. These constraints, both technical and organizational, result in fragmented data collection systems and unequal capacity among stakeholders to analyze and use data effectively. Efforts towards data integration are hampered by sectoral imbalances, particularly between human and animal health sectors in addition to the lack of tools for collecting and centralizing data in environmental sector. A similar situation is denoted in several sub-Saharan African countries, constraining the performance of surveillance systems (17–19).

Stakeholders report difficulties in accessing and using digital platforms often linked to limited network coverage and a lack of suitable equipment. Effectiveness of data-sharing platforms does not depend only on how they are set up but also on the ability of those who are involved in their appropriate use (20).

This study also highlights the importance of improving data-sharing mechanisms between public and private sectors. In Senegal, as in other African countries, private sector and NGOs contribution to zoonosis surveillance remains under-exploited, despite their regular field visits and quality of the data they can provide. The initiatives of these stakeholders are hampered by a lack of a framework to promote exchange and integration of their data. This compartmentalization limits representativeness of data and weakens the system’s ability to detect early warning signals (21).

Furthermore, zoonotic disease surveillance in Senegal relies on several sectoral systems, using a diverse set of methods, pursuing distinct objectives, while facing distinct priorities and constraints. This unconnected diversity impede data integration and makes it only a secondary concern for stakeholders. Therefore, in order to allow for integration, the reinforcement of existing systems and the gradual building of a shared framework around mutually beneficial stakes are first needed. Such a collaborative framework would have to be defined by stakeholders to take into account the distinct empowerment needs of each partner and avoid superimposing a call for integration among other priorities already competing for scarce human, technical and financial resources. Studies conducted in Vietnam and France already illustrated this need for prior consolidation of existing systems (22).

Similarly, our results show that the disparity in stakeholders’ skills and the work overload contribute to an under-exploitation of data. These observations corroborate previous studies that highlighted the link between limited training, work overload and low use of data in planning (20–22). The frequent use of paper as a data storage support leads to data loss and delays in their transmission, which decrease surveillance system effectiveness. This constraint combined with under use of data at all levels directly impacts the effectiveness of the system (23, 24). Hence, in order to overcome these challenges, it is essential to strengthen stakeholders’ skills, including those of local communities. The emergence of new technologies, mainly artificial intelligence and big data, opens up promising prospects for advancing zoonotic disease surveillance. In addition to the already mobilized technologies, these tools could contribute to detect and respond to epidemics (25). In Senegal, although needs for more basic improvements have been identified, the introduction of these advanced technologies may be viewed as a potential for leapfrogging, that has already been experienced on the African continent, as in the case of telecommunications.

### 4.2 Implications of stakeholders’ attitudes toward data sharing

Our findings confirm that trust and transparency in data sharing are essential for effective and sustainable cross-sector collaboration, as previously observed in other One Health initiatives. Stakeholder engagement requires building mutual trust across institutions and ensuring that participants perceive clear benefits from collaboration while maintaining their sectoral autonomy (26). However, trust is not enough to ensure a reliable framework for data sharing. Data are often considered as a strategic resource and power. As a result, some stakeholders are reluctant to share them, fearing a loss of control, a lack of recognition for their contribution or even inappropriate uses.

This posture can be analyzed from the perspective of strategic actor theory, which stipulates that the asymetry of information represents power that stakeholders mobilized to reinforce their position within an organization or pursue their own goals (27, 28). Consequently, control over data becomes a strategic asset, allowing stakeholders to influence decisions and maintain a dominant position. This attitude is reinforced in research and laboratory sectors by the stake of publication, sharing data before publication being perceived as a risk of intellectual expropriation. Thus, the dual use of epidemiological data, for global scientific knowledge production and for direct decision-making generates competing priorities among stakeholders. This tension is amplified by weak collaboration between scientific and administrative staff, each pursuing distinct agendas, which contributes to limiting data sharing.

Furthermore, the lack of clear regulations that define responsibilities and expected benefits, increase reluctance of stakeholders to commit to an ad-hoc sharing arrangement that could be unfavorable to them. This situation highlights the difficulty to reconcile sometimes divergent interests between stakeholders from different sectors. Any collective undertaking relies on harmonization through external mandates or mutual consent (27). In the context of zoonotic surveillance in Senegal the absence of shared regulatory framework hinders either implicit or explicit negotiation on the conditions for data sharing.

Despite these constraints, some stakeholders are motivated to share their data, feeling there are some advantages, either in terms of institutional recognition or public health impact. This aligns with critical analyses that consider that stakeholders continuously adjust their objectives according to opportunities and constraints linked to their environment (25, 26). The motivation in sharing data is even stronger when health workers receive feedback on the outcomes of their reporting efforts. Structured feedback enhances performance and encourages data sharing, as studied in Peru and Tanzania (18, 29). In absence of such feedback, community actors involved in data collection clearly expressed their frustration, weakening their commitment and sense of ownership. It is therefore essential to provide them with regular and meaningful information to keep their commitment over time.

Lastly, cultural and organizational differences between actors create barriers to effective collaboration. Misunderstandings rooted in different professional cultures can affect and complicate data sharing. Therefore, it is essential to strengthen communication channels and promote mutual understanding and trust to overcome these cultural differences (27, 30).

### 4.3 Data sharing governance: inter-sectoral issues and dynamics

In Senegal, inter-sectoral governance of One Health approach is ensured by the One Health Platform, initiated with the support and orientations of international technical and financial partners (TFPs). The One Health Platform is lodged in Prime Minister’s office, which gives it legitimacy to act as a convener mobilizing all ministries. However, despite this institutional legitimacy, doubts have been expressed by stakeholders about whether it is the most appropriate body to steer this governance. More precisely, some actors still advocate that supervision should be under the Ministry of Health while others deem that an independent body is needed in order to avoid any sectoral domination. Previous work has shown that most structures attached to a specific sector such as human health or research are criticized for their lack of impartiality and their difficulty in ensuring effective inter-sectoral coordination (31, 32).

In addition, several initiatives have been implemented to strengthen integrated zoonotic diseases surveillance in Senegal. However, these initiatives have not yet led to the establishment of fully integrated, functional, and sustainable governance mechanisms at national level, mainly due to the lack of a regulatory framework and the limited public financial resources to ensure the continuity and coordination of these systems (20, 31). Hence, the context appears as characterized by a multiplicity of stakeholders, with fragmented, local and temporary initiatives, that the lack of a regulatory framework impedes to gather in a meaningful and viable whole operating at national scale.

Formalizing such a framework is essential to clarify role and responsibilities for stakeholders. It would also help ensure data protection and promote recognition of individual contributions (5, 19).

Furthermore, the role of TFPs in setting up the One Health Platform, with the particular need to comply with International Health Regulations (IHR), leads to some criticisms among national stakeholders. The predominant role of WHO, WHOA, and FAO is sometimes perceived as too prescriptive and some stakeholders regret a mismatch between the priorities set at the international level and local realities (20, 33), but also the lack of national commitment to take over an ownership on these health security stakes. As a result, initiatives for zoonotic control are supported through one-off funding, undermining their viability. This is also seen as a reason why such initiatives are limited to targeted technical activities, mostly in a specific sector, while stakeholders emphasize the need for more integrated and cross-sectoral approaches. Political commitment also remains a major challenge. Frequent changes in political leadership at different levels hinder the operationalization of One Health approach in Senegal. However, it is worth noting that the One Health Platform has shown resilience by maintaining its presence and activities despite political shifts and institutional reorganizations. Nevertheless, such instability still affects its ability to influence lead effectively as described by Zinsstag et al. (6), and confirmed by the results of this study, successful implementation of a data-sharing information system based on One Health approach requires active government involvement and strong political will.

In light of these findings, it becomes evident that strengthening governance mechanisms is essential to ensure integrated data sharing. However, governance alone will not be enough. It is equally essential to build trust between stakeholders and strengthen technical capabilities. Trust fosters transparent collaboration, while technical disparities can reinforce inequalities and perpetuate silos. Therefore, a successful governance framework must integrate strategies that simultaneously build mutual trust and bridge capacity gaps address (34).

In this regard, existing initiatives, such as One Health Platform offer a promising foundation to strengthen collaborative mechanisms. Moreover, collaborative models, such as adaptive and inclusive framework proposed by Leblanc and Williams-Jones (34), based on transparency, consultation, and accountability, can guide the development of sustainable data-sharing systems.

This study allowed us to identify issues related to data sharing useful for zoonotic diseases surveillance in Senegal through interviews with key stakeholders. Lack of new information in last interviews shows that data saturation has been reached. In order to better capture the diversity of stakeholders’ perceptions, we focus our interviews on 61 stakeholders, although the literature indicates that a sample of 15 interviews can be enough to achieve saturation (35). However, a restricted sample may carry a risk of representativeness, particularly if certain stakeholders are underrepresented. Though, we took care to include a representative from each key category, we cannot guarantee that the points of view expressed systematically reflect those of the entire organization, institution or profession involved in zoonotic diseases surveillance in Senegal.

**Figure 2:**
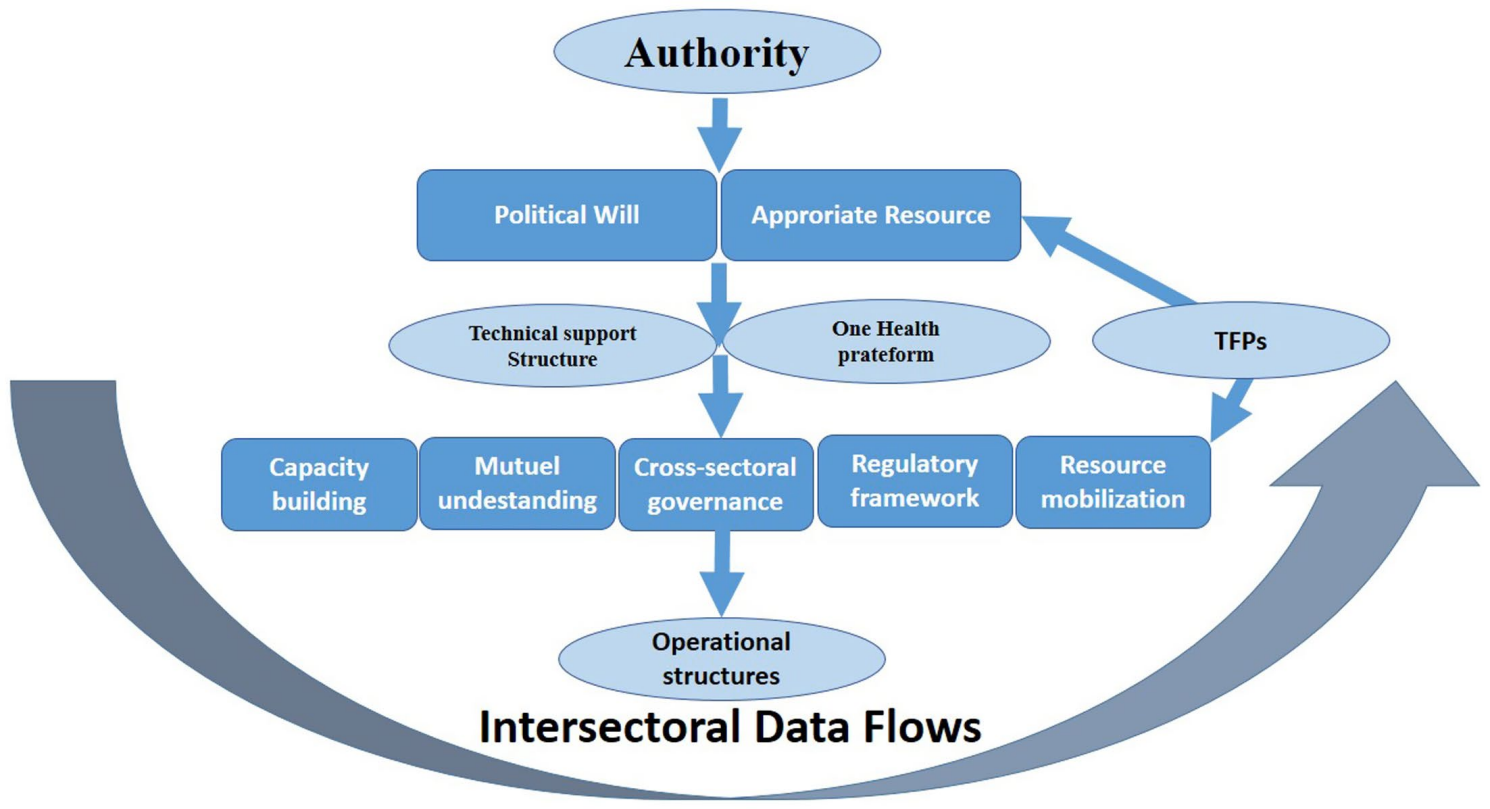
A guidance framework to support the operationalization of zoonotic data sharing using a One Health approach in Senegal.

Based on this situation analysis, we synthesized the themes from our analysis into a governance framework that specifies the functions and roles that participants identified as necessary for sustained intersectoral data sharing (Figure 2). The framework links governance functions, institutional roles, and routine data-sharing practices, with authority providing the foundational political will and resources. It integrates three key dimensions that recurred across interviews: technical and organizational capacity, stakeholder attitudes to sharing, and governance mechanisms.

These dimensions are organized into governance functions that operate together to strengthen routine data exchange. The Technical Support Structure covers infrastructure, skills, and practical assistance needed for reporting, access, and use. Cross-sectoral Coordination and Mutual Understanding address the relational and procedural conditions that sustain collaboration over time. These include trust, incentives, and regular communication and review routines. Regulatory and accountability elements cover mandates, responsibilities, safeguards, and processes for resolving disagreements. These elements reduce perceived risks and clarify expected benefits for participating institutions. Resource mobilization reflects the need for stable financing and the conditions under which external support can reinforce national stewardship. The framework positions these functions as mutually reinforcing rather than sequential. For instance, technical capacity alone cannot enable data sharing without concurrent trust-building and regulatory clarity.

The framework aligns with existing One Health governance approaches by translating high-level principles into governance functions that reflect the constraints reported by participants. In this sense, it operationalizes the implementation expectations emphasized in OHHLEP-related governance guidance, particularly the need to align political commitment, institutional coordination, and system capabilities that enable across-sectoral collaboration (36). It also aligns with health data governance perspectives that emphasize stewardship, clear roles, safeguards, and the capability to use shared data effectively (37).

Unlike frameworks that assume fully functioning systems, this framework explicitly acknowledges contextual constraints and proposes governance adaptations. Its theoretical contribution is therefore deliberately bounded and context-specific. It specifies how established governance and data stewardship expectations can be configured when three constraints consistently shape practice: dependence on external financing, power asymmetries across sectors, and limited technical infrastructure for routine data exchange (38).

This framework should be understood as a governance framework rather than a causal or predictive model. Implementation can be monitored using process indicators associated with each governance function presented in Figure 2. Together, these elements provide an actionable structure for operationalizing intersectoral data flows for zoonotic surveillance in Senegal.

## 5 Conclusion

In Senegal, implementing effective zoonotic diseases surveillance through a One Health approach faces specific challenges. This study identified three major challenges to integrated zoonotic data sharing: limited technical and organizational capacity, contrasting stakeholder attitudes, and fragmented governance. These challenges are compounded by political instability and a strong dependency on external funding. Despite these barriers, some enabling factors were identified, such as the strong motivation of stakeholders, the existence of the One Health Platform, and previous experiences of intersectoral collaboration.

To overcome these barriers and improve epidemic response, a sustainable strategy is needed. This strategy must be based on political will with effective stakeholder engagement. It must also be supported by adequate financial, human, and institutional resources. Furthermore, it should be anchored in a transparent regulatory framework and robust inter-sectoral governance. Building resilience through sustainable governance and robust cross-sectoral collaboration is essential to face emerging zoonotic threats.

## Data Availability

The datasets presented in this article are not readily available because the qualitative datasets generated and analyzed during this study contain sensitive information that could compromise participant confidentiality. In accordance with institutional and ethical data protection policies, the full datasets cannot be shared publicly. However, anonymized excerpts and the coding framework may be made available from the corresponding author upon reasonable request, provided that the request aligns with participant confidentiality and data protection regulations. Requests to access the datasets should be directed to Diouf Khady, khady.diouf@isra.sn.
